# Enhancing cardiac assessments: accurate and efficient prediction of quantitative fractional flow reserve

**DOI:** 10.3389/fbioe.2025.1438253

**Published:** 2025-01-27

**Authors:** Arshia Eskandari, Sara Malek, Alireza Jabbari, Kian Javari, Nima Rahmati, Behrad Nikbakhtian, Bahram Mohebbi, Seyed Ehsan Parhizgar, Mona Alimohammadi

**Affiliations:** ^1^ Department of Mechanical Engineering, K.N. Toosi University of Technology, Tehran, Iran; ^2^ Rajaie Cardiovascular Medical and Research Center, Iran University of Medical Sciences, Tehran, Iran

**Keywords:** coronary artery disease, fractional flow reserve, myocardial infarction, noninvasive imaging, computational fluid dynamics, virtual surgery

## Abstract

**Background:**

Obstruction within the left anterior descending coronary artery (LAD) is prevalent, serving as a prominent and independent predictor of mortality. Invasive Fractional flow reserve (FFR) is the gold standard for Coronary Artery Disease risk assessment. Despite advances in computational and imaging techniques, no definitive methodology currently assures clinicians of reliable, non-invasive strategies for future planning.

**Method:**

The present research encompassed a cohort of 150 participants who were admitted to the Rajaie Cardiovascular, Medical, and Research Center. The method includes a three-dimensional geometry reconstruction, computational fluid dynamics simulations, and methodology optimization for the computation time. Four patients are analyzed within this study to showcase the proposed methodology. The invasive FFR results reported by the clinic have validated the optimized model.

**Results:**

The computational FFR data derived from all methodologies are compared with those reported by the clinic for each case. The chosen methodology has yielded virtual FFR values that exhibit remarkable proximity to the clinically reported patient-specific FFR values, with the MSE of 6.186e-7 and R2 of 0.99 (p = 0.00434).

**Conclusion:**

This approach has shown reliable results for all 150 patients. The results are both computationally and clinically user-friendly, with the accumulative pre and post-processing time of 15 min on a desktop computer (Intel i7 processor, 16 GB RAM). The proposed methodology has the potential to significantly assist clinicians with diagnosis.

## 1 Introduction

The formation of blockage in the left anterior descending coronary artery (LAD) is a frequent phenomenon and a prominent independent predictor of mortality (Mohammad et al., 2022; Perry et al., 2021). Non-invasive diagnostic strategies are being pursued to identify stenotic lesions within the LAD artery (Vardhan et al., 2021). Fractional flow reserve (FFR) is a hemodynamic index used to assess coronary artery disease, and values below 0.8 signify the presence of myocardial ischemia, necessitating revascularization interventions (Feng et al., 2022; Zhang et al., 2023; Zhang et al., 2020).

Recent advancements have integrated computational fluid dynamics with medical imaging data to obtain FFR. The non-invasive method known as Quantitative Flow Ratio (QFR) uses computational algorithms to estimate FFR. Several studies have investigated the efficacy of QFR in assessing coronary artery disease, demonstrating promising potential as an alternative to pressure wire measurements (Chandola et al., 2021; Milzi et al., 2021; Zhang et al., 2022; Tar et al., 2018; Westra et al., 2018). In 2018, a study was conducted Kołtowski et al. (2018) to examine the precision of QFR and found an accuracy of 85.4% for the QFR index when enrolling a cohort of 268 patients. In 2019, a study was conducted Smit et al. (2019) to determine whether using QFR was feasible for referring patients to the FFR procedure. The study effectively identified FFR values below 0.8 with a precision of 86% for a total of 290 patients. However, the current level of precision makes this method unsuitable for replacing pressure wire. In 2019, an investigation Watarai et al. (2019) explored the applicability of QFR for the assessment of coronary stenosis. Their comparative analysis suggests a promising capability for evaluating myocardial ischemia within clinical contexts, with a sensitivity of 85% and a specificity of 83% for QFR. Similarly, another research Tanigaki et al. (2019) investigated the diagnostic performance of QFR derived from coronary angiography, using FFR as the reference standard. They found that the accuracy for predicting FFR values below 0.8 using QFR was 58%. Despite the favorable correlations demonstrated by Quantitative FFR approaches with pressure wire measurements, these methodologies still lack the required accuracy for clinical implementation.

Accurate estimation of characteristics of the vessel is crucial for accurately predicting the pressure gradient within the artery and hence calculation of QFR. The Windkessel model explains compliance and resistance characteristics in the arterial system within cardiovascular physiology and establishes a connection between pressure and flow in the cardiovascular system (Westerhof et al., 2009; Duanmu et al., 2018; Duanmu et al., 2019). Furthermore, by utilizing Windkessel models, the capability to generate outputs that conform well to experimental observations becomes possible (Kissas et al., 2020). Different Windkessel models with varying complexity exist, with higher complexity providing more accurate results. However, increased computational burden should be taken into account. Additionally, it's important to note that arterial characteristics vary among patients (Zhou et al., 2019; Mariscal-Harana et al., 2020); Nonetheless, there remains a gap in exploring the integration of patient-specific (P.S.) arterial characteristics into research endeavor.

This study aims to develop an FFR estimation technique that outperforms previous approaches. Prior investigations have approached this problem as a classification task with predefined thresholds (e.g., classifying FFR below/above 0.8), which fails to offer reliable insights into the methodology’s effectiveness. In contrast, the present study directly estimates the FFR value and assesses performance by comparing it to the actual value, resembling a regression task. Furthermore, the integration of P.S. arterial characteristics for accurate predictions will be explored to fill the existing gap in this field. Additionally, the current investigation offers valuable hemodynamic information as a predictive tool to identify the atherosuseptible sites of arteries, which can provide beneficial insight for surgical treatments. A total of 150 patients, two methodologies, and an array of Windkessel models were examined, with the goal of evaluating the efficacy of each methodology. The proposed methodology demonstrated notable conformity to the experimental data, promising a high level of efficacy in estimating FFR.

## 2 Methods

### 2.1 Research population and acquired data

This study includes 150 participants from the Rajaie Cardiovascular, Medical, and Research Center. Four of them are reported herein for showcasing the results. All participants gave their informed consent, and the study was approved by the Iran National Committee for Ethics in Biomedical Research with the ethics approval number IR. IUMS.FMD.REC.1401.246. To ensure impartiality and avoid bias, four patients for FFR analysis were randomly selected. The hospital provided coronary angiography image stacks and corresponding FFR data for each participant. The FFR dataset included the FFR value, proximal arterial pressure waveform, and distal pressure waveform for each patient.

### 2.2 Geometry reconstruction

**FIGURE 1:**
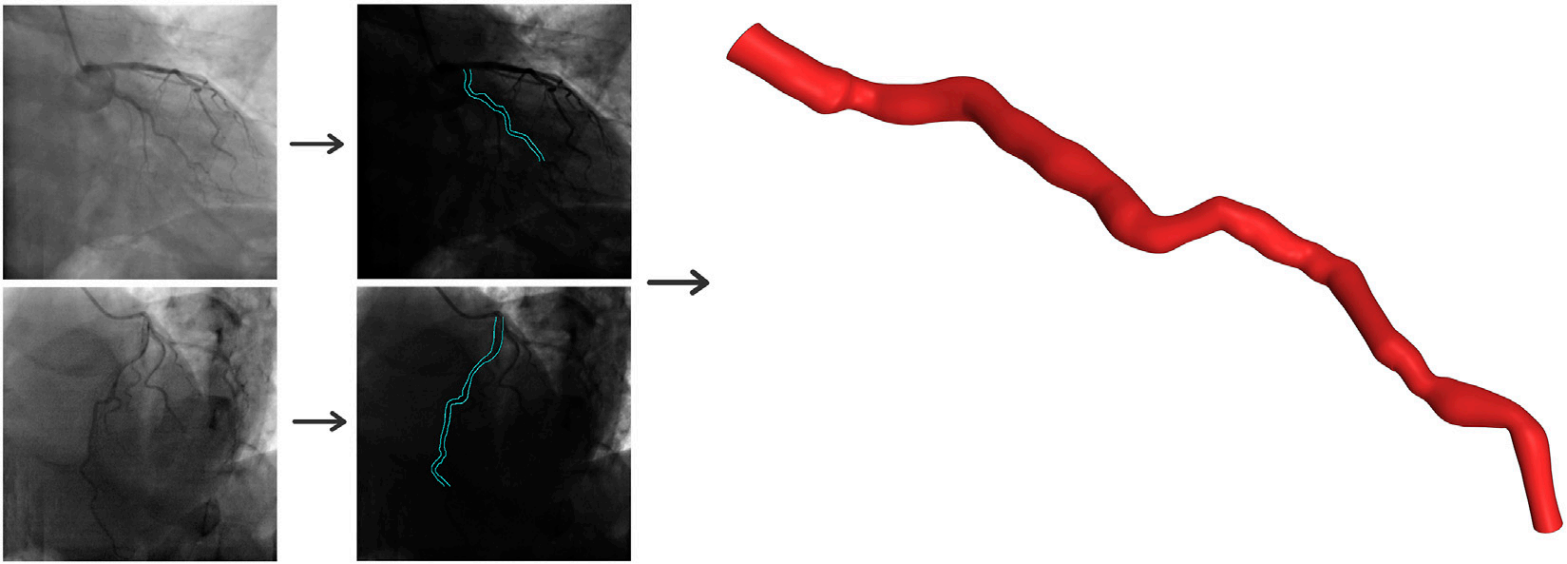
Three-dimensional reconstruction of the coronary artery using two angiographic projections at least 25° apart.

The three-dimensional structure of the LAD artery is reconstructed using two distinct angiographic perspectives at the end of the cardiac cycle. Edge detection techniques and 3D mapping algorithms are utilized to transform the views into a 3D representation. Figure 1 provides a visual guide for the geometry reconstruction process from angiography images.

### 2.3 Computational mesh generation

The ANSYS Meshing (ANSYS Inc., Canonsburg, PA, United States) was employed to discretize the domain, resulting in a mesh comprising approximately 90,000 tetrahedral cells, five prismatic layers with a 1.2 growth ratio to mitigate computational inaccuracies in the vicinity of the boundary. Mesh independence analyses were performed, wherein three grids were constructed: a coarse mesh of approximately 45,000 cells and a fine mesh of approximately 180,000 cells. The difference between the coarse and medium grids is below the 3.1% threshold. Additionally, the variability between the medium and fine grids is confined to 0.92%. Consequently, the medium mesh is chosen as the compromise to reduce computational expenses.

### 2.4 Computational fluid dynamics

ANSYS-CFX software was used to solve the equations of continuity and Cauchy, represented as Equations 1, 2 respectively. The governing equations incorporate a second-order backward Euler scheme and the discretization of each cycle into 1,000 steps. Additionally, the maximum allowable residual mean square errors are defined at 1 × 10^−4^.

$$\nabla .\vec{u}=0$$ (1)

$$\rho \frac{D\vec{u}}{Dt}=\rho f+\nabla \cdot \bar{\sigma }$$ (2)

In the given equations, $\vec{u}$ symbolizes the velocity of the fluid, while $\bar{\sigma }$ , ρ and f symbolize the stress tensor, density, and a vector containing all of the accelerations caused by body forces, respectively.

### 2.5 Blood properties

Blood is modeled as an incompressible fluid with a density of $1060kg/m^{3}$ and is considered to be non-Newtonian. Its dynamic viscosity is modeled by Carreau–Yasuda model, described in Equation 3. Within this model, the viscosity ( μ ) depends on: shear rate ( $\gamma^{\prime }$ ), Carreau–Yasuda zero shear viscosity ( $\mu_{0}$ ), Carreau–Yasuda infinite shear viscosity $\left(\mu_{\infty }\right)$ , Yasuda exponent ( a ), Carreau–Yasuda Power Law Index ( m ), and Carreau–Yasuda time constant ( $\lambda_{CY}$ ).

$$\mu ={\left(\mu_{0}-\mu_{\infty }\right)\left({\left(1+\lambda_{\text{CY}}\gamma^{\prime }\right)}^{a}\right)}^{m-1/a}+\mu_{\infty }$$ (3)

where $\mu_{0}=22\times 10^{-3}\;Pa\;s$ , $\mu_{\infty }=2.2\times 10^{-3}\;Pa\;s$ , a=0.644 , n=0.392 , $\lambda_{CY}=0.110\;s$ . The parameters utilized in this study were derived from a research conducted 2021 (He et al., 2021).

### 2.6 Boundary conditions

The Windkessel (lumped-parameter or 0D) model describes various physical phenomena using electrical circuits. Five 0D models have been employed to describe the boundaries of the computations. The first model is a one-element Windkessel model with one resistance ( R ). Equation 4 describes this model.

P=RQ (4)

where P represents the pressure drop across the circuit, and Q represents the flow rate. The second model is a two-element model; with a capacitance ( C ) added to the previous model. Equation 5 describes the model.

$$P=RQ-RC\frac{dP}{dt}$$ (5)

The third and fourth models are both three-element models. One model consists of two resistance components and a capacitance, while in the other model, the resistance has been replaced by an inductance ( L ). Equations 6, 7 describe the three-element models.

$$P=\left(R_{1}+R_{2}\right)Q-R_{2}C\frac{dP}{dt}+R_{1}R_{2}C\frac{dQ}{dt}$$ (6)

$$P=RQ+RC\frac{dQ}{dt}+RCL\frac{d^{2}Q}{dt^{2}}-RC\frac{dP}{dt}$$ (7)

The fifth model is a four-element Windkessel model. This model comprises two resistance components, one capacitance and one inductance component. Equation 8 describes this model.

$$P=\left(R_{1}+R_{2}\right)Q+{R_{1}R}_{2}C\frac{dQ}{dt}+R_{2}CL\frac{d^{2}Q}{dt^{2}}-R_{2}C\frac{dP}{dt}$$ (8)

**FIGURE 2:**
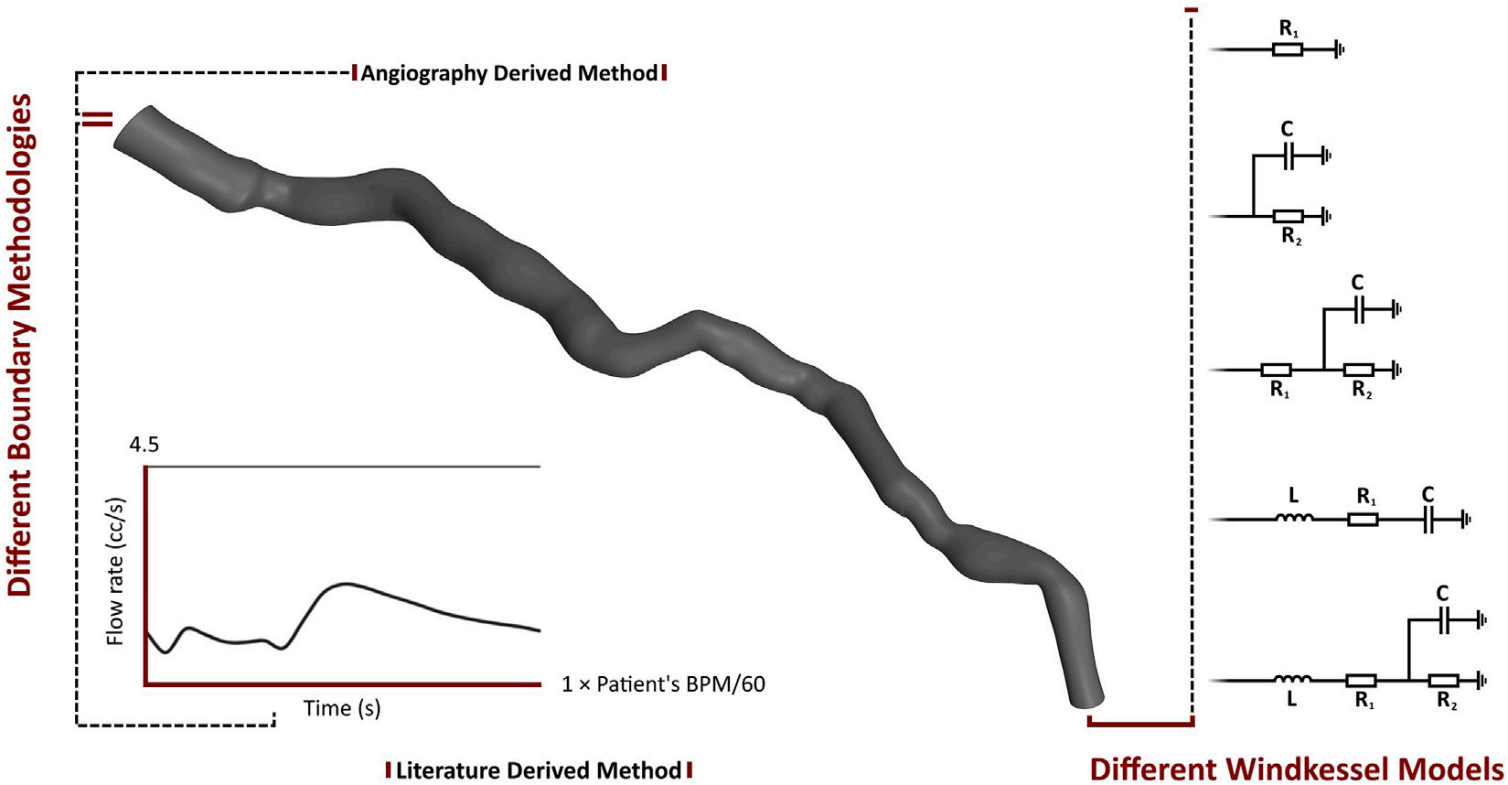
Boundary conditions of the different cases for each patient.

The parameters of the 0D models were calibrated for each patient. In addition to various Windkessel models, this study utilizes two methodologies: AngioBC and LiteratureBC. AngioBC involves patient-specific inlet pressure obtained from Angiography, while LiteratureBC employs an inlet flow rate derived from literature (Kim et al., 2010), calibrated based on the cardiac cycle time of the patients. Figure 2 summarizes the boundary conditions established for the different cases developed for each patient. The Shear-Stress Transport (SST) model has been chosen as the turbulence model for the analysis, considering the low magnitude of 1% in intensity.

### 2.7 Measurement of hemodynamic parameters

This investigation examines the FFR and time-averaged wall shear stress (TAWSS). As the effect of wall shear stress (WSS) magnitude in atherosclerosis formation has long been established, TAWSS has been introduced for the prediction of future blockage or the advancement of infarction within the arteries. Low TAWSS can trigger endothelial dysfunction and inflammation, leading to the formation of atherosclerotic plaques. Areas of low TAWSS are associated with vulnerable plaques that are more prone to rupture. Plaque rupture can lead to the formation of thrombosis and cause acute cardiovascular events. Changes in TAWSS can indicate alterations in blood flow patterns within the artery. Disturbed flow patterns, such as recirculation zones or flow separations, can promote plaque development and instability. TAWSS analysis can provide valuable insights into patient-specific risk factors for arterial diseases. By combining TAWSS data with other clinical information, clinicians can better assess an individual’s risk of developing arterial blockages or experiencing adverse cardiovascular events (Moerman et al., 2022; Mazzi et al., 2021; Maher et al., 2021). By integrating wall shear stress (WSS) at every nodal point throughout a complete cardiac cycle, TAWSS is calculated. TAWSS is mathematically represented by Equation 9.

$$TAWSS=\frac{1}{T}\;\int_{0}^{T}\left|WSS\right|dt\;$$ (9)

where the variable T represents the duration of a complete cardiac cycle, while t refers to a moment within the timeframe. For the assessment of FFR, the distal pressure downstream of the stenosis is divided by the proximal pressure upstream of the stenosis. This relationship is expressed in Equation 10.

$$FFR=\frac{P_{Distal}}{P_{Proximal}}$$ (10)

where in $P_{Distal}$ and $P_{Proximal}$ represent distal and proximal pressures, respectively.

### 2.8 Performance metrics

The mean absolute error (MAE), mean squared error (MSE), root mean squared error (RMSE), R-squared (R2) and Explained Variance Score (EVS) metrics are examined to evaluate the performance of each methodology. MAE measures the average absolute difference between the predicted values and the actual values, described in Equation 11:

$$MAE=\frac{1}{n}\sum_{i=1}^{n}\left|y_{i}-\hat{y_{i}}\right|$$ (11)

n is the number of observations, $y_{i}$ is the actual value, and $\hat{y_{i}}$ is the predicted value. MSE measures the average of the squares of the errors or deviations (Equation 12).

$$MSE=\frac{1}{n}\sum_{i=1}^{n}{\left(y_{i}-\hat{y_{i}}\right)}^{2}$$ (12)

RMSE is the square root of the MSE, providing an interpretation in the same unit as the target variable (Equation 13).

$$RMSE=\sqrt{MSE}$$ (13)

R2 score represents the proportion of the variance in the dependent variable that is predictable from the independent variables, expressed in Equation 14.

$$R2=\frac{\frac{1}{n}{\sum_{i=1}^{n}\left(y_{i}-\hat{y_{i}}\right)}^{2}}{\frac{1}{n}{\sum_{i=1}^{n}\left(y_{i}-\bar{y}\right)}^{2}}$$ (14*)*

$\bar{y}$ is the mean of the observed data. EVS indicates the proportion of the variance in the dependent variable that is explained by the independent variables (Equation 15).

$$EVS=\frac{Var\left(y-\hat{y}\right)}{Var\left(y\right)}$$ (15)

$Var\left(y-\hat{y}\right)$ is the variance of the residual, and $Var\left(y\right)$ is the variance of the true values.

## 3 Results

### 3.1 Pressure comparison in the windkessel models

**FIGURE 3:**
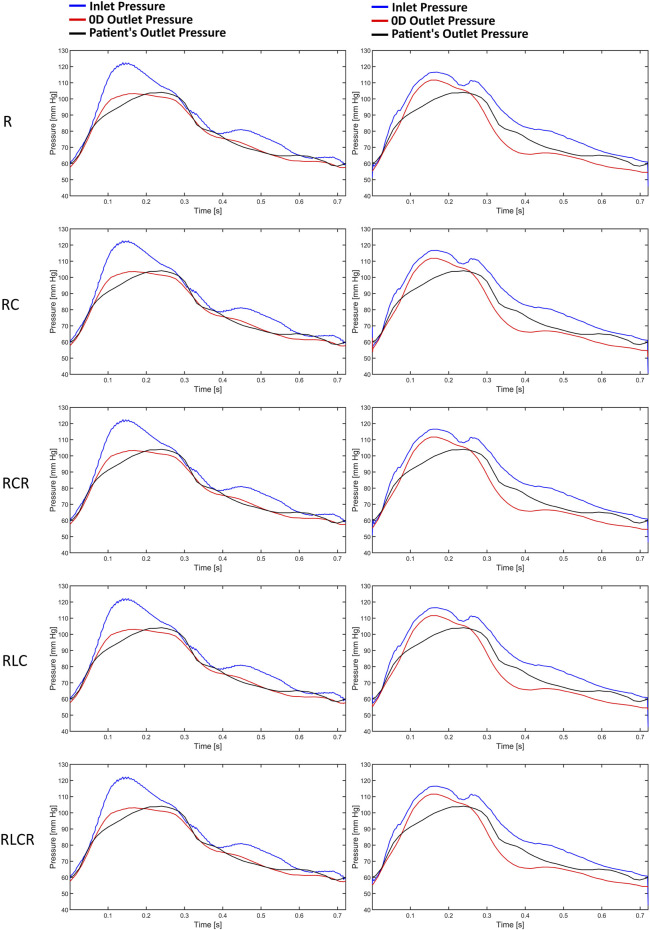
The pressure waves of the Windkessel configurations for patient 1. Left column: AngioBC-0D. Right column: LiteratureBC-0D.

Figure 3 illustrates the pressure waves generated by the Windkessel models for the first patient. It can be observed that the pressure waves of the various Windkessel models’ techniques are similar throughout different outlet boundary designations. The actual peak-systolic pressure is lagged compared to the estimated peak systolic Windkessel pressure. The pressure waves during the diastolic phase fluctuate differently in all models. In the LiteratureBC method, the distal pressure waves have reached the peak at a similar time but lagged from the expected pressure points. Moreover, the outlet pressure is over-predicted just before the peak systole and under-predicted after the peak. The inlet pressure waves of the AngioBC-0D and the LiteratureBC-0D models do not match; the AngioBC-0D models have a higher peak with the fluctuations of the dicrotic notch for the LiteratureBC models.

### 3.2 TAWSS comparison in the windkessel models

**FIGURE 4:**
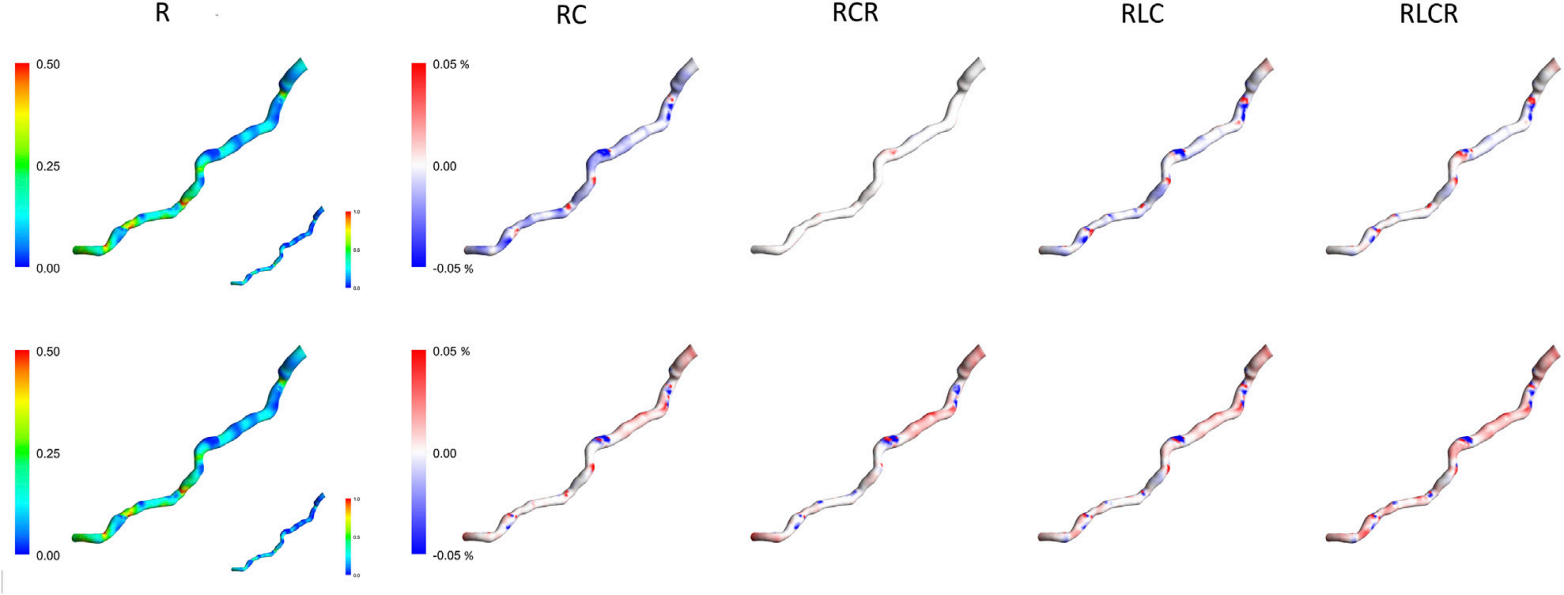
TAWSS contours of the Windkessel configurations for patient 1. Top row: AngioBC-0D. Bottom row: LiteratureBC-0D. TAWSS: time-averaged wall shear stress.

Figure 4 compares the TAWSS for the first patient using two methods and various lumped parameter models. The first column shows the TAWSS estimation for the singular resistance lumped model, with subsequent columns illustrating relative differences with the incorporation of different lumped elements. The TAWSS distribution is similar within methodologies. Different Windkessel parameters produce almost identical TAWSS estimations. The difference in TAWSS estimation by Windkessel models is at most 0.05%. The downstream segment exhibits higher TAWSS, while the upstream displays lower relative TAWSS in both methods. The Windkessel model approximations converge closely towards the downstream region, except for the AngioBC-RC model. As the Windkessel model gets more complex, AngioBC-0D models yield lower TAWSS than the R model. However, in the LiteratureBC-0D approach, increasing the complexity results in higher TAWSS, except for the AngioBC-RCR model. Overall, the Windkessel models show similarities in the patterns of disparities. The LiteratureBC-RLCR model varies the most compared to the R model. Windkessel evaluations are lower in value in the center of the artery compared to the AngioBC-R model.

### 3.3 Pressure comparison in different BC approaches

**FIGURE 5:**
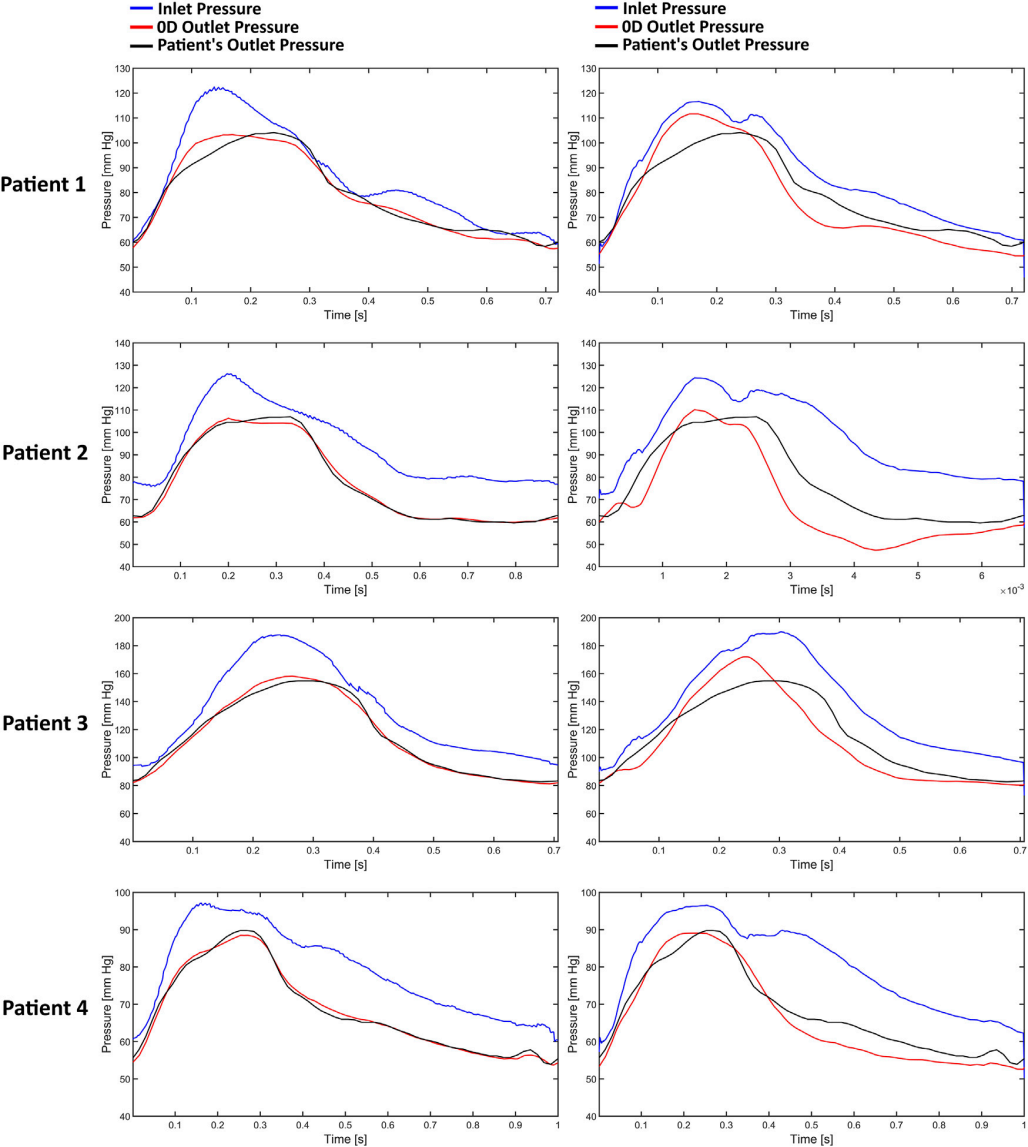
The pressure waves of each patient from the one resistance model. Left column: AngioBC-0D. Right column: LiteratureBC-0D.

Figure 5 presents a demonstration of the pressure waves generated by the AngioBC-0D method (left column) alongside the LiteratureBC-0D models (right column) from the singular resistance model for all four patients. Windkessel models with lumped parameters and Angio-derived conditions at the inlet generate similar pressure waves regardless of the outlet boundary method. Literature-derived flow models also show agreement between the outlet and anticipated pressure. FFR-measured peak systolic pressure lags behind estimated Windkessel peak systolic pressure. All models show noticeable variances in outlet pressure wave fluctuations during the systolic phase. Comparing patient-specific and literature-derived boundary conditions reveals similar peak times in distal pressure waves but with discrepancies in anticipated pressure points. Literature-derived models overpredict outlet pressure just before peak systole and underpredict the pressure post-peak. Additionally, inlet pressure waves in AngioBC-0D and LiteratureBC-0D models differ, with the latter showing higher peaks and dicrotic notch. In LiteratureBC, outlet pulse pressure surpasses both FFR data and pulse pressure from the AngioBC model. Patient 1 showed a small gap between distal and proximal pressure waves, while Patient 2 had a larger disparity. The other two patients fell in between these extremes.

### 3.4 TAWSS comparison in different BC approaches

**FIGURE 6:**
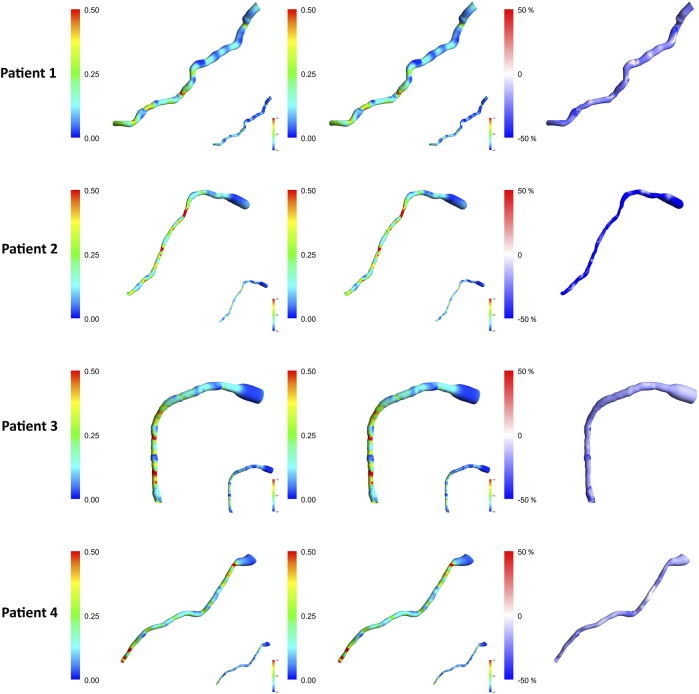
TAWSS contours for each of the patients from the R model. Left column: AngioBC-0D. Middle column: LiteratureBC-0D. Right column: the difference between the two methods. TAWSS: time-averaged wall shear stress.

Figure 6 demonstrates the TAWSS contours of the one resistance model for the Angio-derived boundary model (left column), the Literature-derived flow inlet boundary (middle column), and the discrepancy between these models for each patient within the cohort (right column). Comparable TAWSS distributions are discernible in both boundary techniques. The upstream portion of the geometry has yielded diminished TAWSS magnitudes, whereas the downstream section of the artery showcases elevated relative values across both methodologies. The maximum relative TAWSS occurs downstream of LAD, while the minimum occurs at every patient’s topmost section of the artery. In the most constricted regions of the artery, an elevation in TAWSS is observable across all models. Furthermore, the LiteratureBC produces relatively lower TAWSS than the AngioBC.

### 3.5 Expanded TAWSS analysis across a larger patient cohort

**FIGURE 7:**
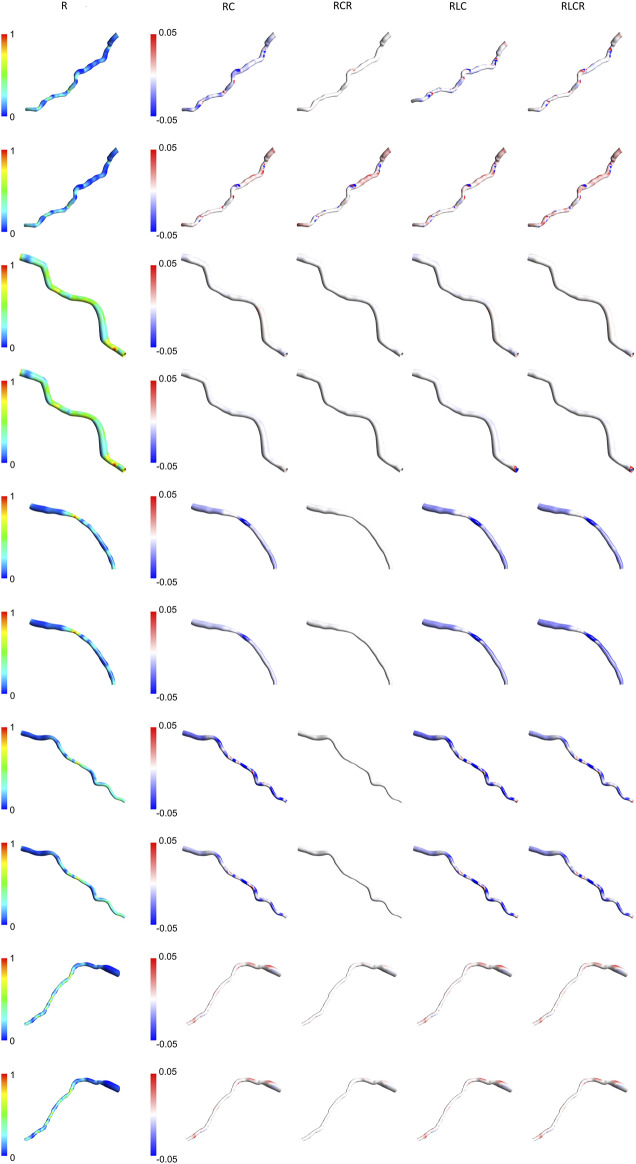
TAWSS contours of patients 1–5 for the R Windkessel model, with deviations from other 0D models. The top row shows AngioBC, and the bottom row shows LiteratureBC.

**FIGURE 8:**
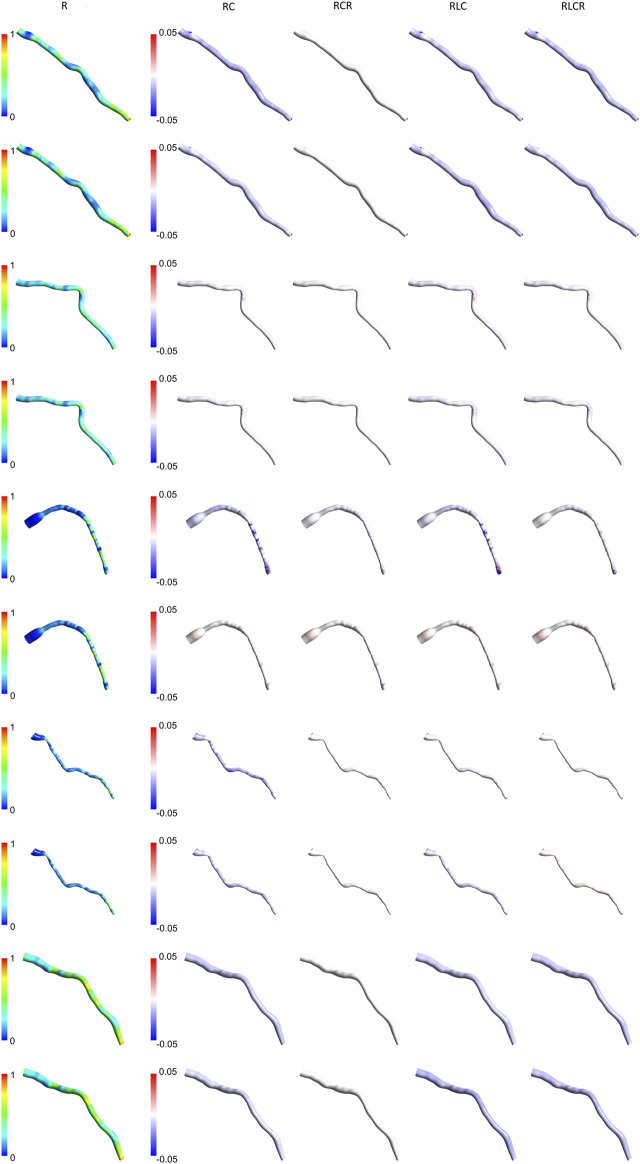
TAWSS contours of patients 6–10 for the R Windkessel model, with deviations from other 0D models. The top row shows AngioBC, and the bottom row shows LiteratureBC.

Figures 7, 8 present the normalized TAWSS contours for the R Windkessel model across 10 patients, along with the deviations from other 0D models (RC, RCR, RLC, RLCR) for each corresponding patient. For each patient, the top row represents results from the AngioBC methodology, while the bottom row corresponds to the LiteratureBC methodology, providing an analysis of a larger cohort. As shown in the figures, the variations in TAWSS estimations across different Windkessel parameters are negligible, with a maximum discrepancy of only 0.05%, similar to observations in the previous sections. In both methodologies, higher TAWSS values are consistently observed in the downstream arterial segments, while the upstream regions exhibit comparatively lower TAWSS magnitudes. The results between the two boundary condition methods are closely aligned, showing only marginal differences, and the contour distributions are nearly identical. The highest relative TAWSS values are consistently located downstream of the LAD, while the lowest values are found at the topmost sections of the arteries in all patients. Notably, the most constricted regions of the arteries exhibit a pronounced increase in TAWSS across all models, with relative TAWSS increasing as the degree of narrowing increases. This observation is consistent with findings from previous sections.

### 3.6 The validation and the QFR

**FIGURE 9:**
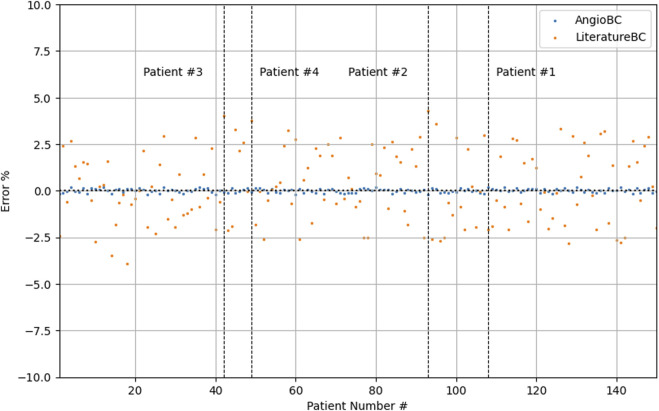
Distribution of error in methodologies.

Table 1 documents the performance metrics for both of the methodologies, while Table 2 expounds upon the one-element cases examined within this study. The first patient had the highest FFR among examined subjects, while the second patient had the lowest. The QFR values obtained from the AngioBC closely match the patient-specific FFR values, and the error is not statistically significant (p = 0.00434), with the MSE of 6.186e-7 and R2 of 0.99. The LiteratureBC approach, although not perfectly aligned with FFR, yielded similar results (p = 0.12715) with the MSE of 2.188e-4 and R2 of 0.97. Figure 9 visualizes the distribution of errors in methodologies.

**TABLE 1:** Performance metrics of the methodologies.

Method	MSE	RMSE	MAE	R2	EVS
AngioBC	6.18647e-07	0.00078	0.00068	0.99992	0.99992
LiteratureBC	0.00021	0.01479	0.01263	0.97389	0.97424

EVS, explained variance score; MAE, mean absolute error; MSE, mean squared error; R2, R-squared; RMSE, root mean squared error.

**TABLE 2:** FFR and QFR data.

Patient #	P.S.-FFR	Literature BC-R	Error % (P.S. vs. literature BC-R)	Angio BC-R	Error % (P.S. vs. angio BC-R)
1	0.91663	0.88936	2.9750%	0.91795	0.1440%
2	0.80960	0.77476	4.3026%	0.81121	0.0901%
3	0.85692	0.82250	4.0167%	0.85771	0.0921%
4	0.85985	0.82741	3.7727%	0.86058	0.0848%

BC, boundary condition; FFR, fractional flow reserve; P.S., patient specific.

## 4 Discussion

The findings suggest that different lumped parameter boundaries had only marginal variances in FFR estimations. Despite the increased precision of using a 4-element model (Alimohammadi, 2018; Shad et al., 2021; Xing et al., 2023), FFR estimations remained relatively consistent. The slight increase in accuracy might not be justify the additional computational cost of the simulation, as the 1-element model already provides a highly accurate estimation of FFR. The peak systolic pressure measured by FFR at the outlet is delayed compared to the estimated Windkessel peak systolic pressure and the peak systolic pressure measured at the inlet. Under normal circumstances, the pressure wave propagates swiftly and seamlessly through the arterial wall. However, when confronted with a constriction or blockage, it decelerates, inducing a temporal lag in the pressure’s transit time to a specific arterial location (Khader et al., 2016; McConkey et al., 2019). This indicates that although the presented 0D models could estimate the pressure accurately, they cannot consider the lag created by the blockage, as 0D models only provide an approximation for the downstream of the vessel (Pfaller et al., 2022). This has caused fluctuations and overprediction of pressure prior to the peak systole, and underprediction of this pressure after the peak. Simulations can differ from individual patient data due to non-specific inlet boundary, the mismatch between AngioBC and LiteratureBC, and the presence of a dicrotic notch. Even with customized LiteratureBC flow, the absence of actual patient data leads to some degree of error, as the inlet boundary alters the vessel’s hemodynamics (Khader et al., 2016; Pfaller et al., 2022; Vanden Eynden et al., 2018; Madhavan and Kemmerling, 2018).

The TAWSS distribution pattern is similar for different Windkessel element combinations (Figure 4) due to the shape of the vessel. The magnitude is linked with the boundaries, as observed by other scholars (Wang et al., 2021; Fandaros et al., 2023). Results from different boundaries (R-RC-RCR-RLC-RLCR) were almost unified due to similar outlet boundary levels. This suggests negligible effects of other parameters in the LAD artery. Other researchers have also reported that secondary lumped parameters of this artery generally exhibit lower values (Westerhof et al., 2009). Noticeably magnified levels of TAWSS have been discernible downstream of simulations due to the visible narrowing of the vessel. Further tapering results in increased TAWSS levels, as reported in various studies (Malek et al., 2025; Qiao et al., 2019; Wang et al., 2019; Chen et al., 2016).

The Windkessel models show some fluctuations in results without any apparent correlation. Some models align well with the R model in the AngioBC method but perform average with the LiteratureBC approach. This indicates the significance of different lumped parameters under dissimilar conditions, although, in this research, it can be shown that the effects of the secondary 0D elements on LAD are minimal. Other authors have also reported this significance (Westerhof et al., 2009; Ojeda et al., 2014). At an area near the vessel’s center, the 0D models with secondary parameters mostly concluded a relatively decreased TWASS estimation; even though this discrepancy is minuscule, it points to inadequacies of the R model in providing the most detailed estimations. As mentioned previously, with the increase in complexity of the 0D system, it is expected that the level of accuracy will also increase, as shown by other scholars (Alimohammadi, 2018), which can be seen in the results of this study. However, for arteries with minimal compliance, the differences are negligible.

The Windkessel model corresponds precisely to the outlet of the vessel when compared to the AngioBC model and FFR measurements, indicating high precision in its tuning (Alimohammadi, 2018). The Literature-derived flow model has also evinced a remarkable concordance between the simulation and invasively acquired outlet pressures, again due to the tuning accuracy of the lumped model parameters. Although compared to the AngioBC model, the output of the LiteratureBC-0D method has demonstrated a noticeable divergence from the invasive FFR data. This points to the importance of the inlet boundary condition for achieving accurate results (Skopalik et al., 2021). Outlet pressure fluctuations in the systolic phase have caused a mismatch in peak systole’s timepoint, resulting in over/underprediction of pressure in systole. Lumped models are more precise in the diastolic phase than the systolic phase, as reported by other investigations (Westerhof and Westerhof, 2017). Moreover, the utilization of the LiteratureBC led to inconsistency between the inlets of two models due to an inflated inlet pressure with a dicrotic notch. This again underscores the significance of boundary conditions for precise computational analyses (Westerhof et al., 2009; Skopalik et al., 2021). The LiteratureBC method has also displayed an overestimated pulse pressure. The literature-derived flow was responsible for this error due to the constant 0D element values. This means the flow may not have been optimal for every patient in this cohort, highlighting the importance of patient-specific boundaries (Westerhof et al., 2009; Skopalik et al., 2021). The LAD’s inlet and outlet for each patient had a distinct disparity due to the influence of the distance between these pressure waves. Hence, the patient with the most significant disparity possessed the lowest FFR value, and *vice versa*, which aligns with the definition of FFR (Xue et al., 2023; Garcia et al., 2019; Ahn et al., 2017; Merkus et al., 2021; Addison et al., 2023).

The TAWSS contours from both AngioBC and LiteratureBC methods are remarkably similar due to the distribution of wall shear being a function of the geometry and the blood characteristics (Wang et al., 2021). Higher TAWSS levels have been reported downstream of the artery due to narrowing in the downstream area of the LAD artery (Dadras et al., 2023). TAWSS increase in tapered regions of the vessel could indicate the existence and the possibility of advance of a blockage in the artery (Hernández-López et al., 2021; Azar et al., 2019). The LiteratureBC model shows lower TAWSS than the AngioBC model. Fluctuations in results are due to pressure drop, which significantly affects TAWSS approximations (Antonuccio et al., 2021).

The AngioBC approach has yielded FFR estimations that exhibit remarkable proximity to the patient-specific FFR values (p = 0.00434) with the MSE of 6.186e-7 and R2 of 0.99. Maximum error percentage of 0.1440% was noticeable in the first patient. LiteratureBC-0D models also show strong agreement with the experimental data (p = 0.12715) with the MSE of 2.188e-4 and R2 of 0.97. Moreover, the validity of the 3D geometry reconstruction technique is substantiated by the notable levels of consistency and performance observed within the results of this approach. Although the LiteratureBC-0D may not achieve absolute correspondence with the AngioBC-0D model, the outcomes have yielded comparable findings, and the outcomes exhibited remarkable efficacy. Despite its relative inferiority in performance when compared to the alternative model, this particular framework nevertheless renders a commendable apparatus for estimating FFR.

## 5 Limitations

Similar to methodologies employed in other studies, initial catheterization is necessary for geometry reconstruction by introducing contrast agents into the vessel. However, there is no need to extend the pressure wire within the artery. Both methods are constrained by geometry, but the first method is additionally restricted by the data obtained from the initial catheterization. This implies that if advancements in non-invasive angiography imaging techniques emerge, the first approach would still be limited to data from catheterization. On the other hand, the second approach is not constrained by this data and remains viable; therefore, it would still be capable of providing highly precise estimations of FFR. Another limitation of the current study is the lack of long-term evaluations to assess the method’s performance over time and in diverse clinical settings. While the methodology has shown promising results, further studies are needed to confirm its reliability in real-world, dynamic conditions. This study, however, provides a strong foundation for future research by demonstrating the method’s potential within the controlled parameters analyzed.

## 6 Conclusion

**FIGURE 10:**
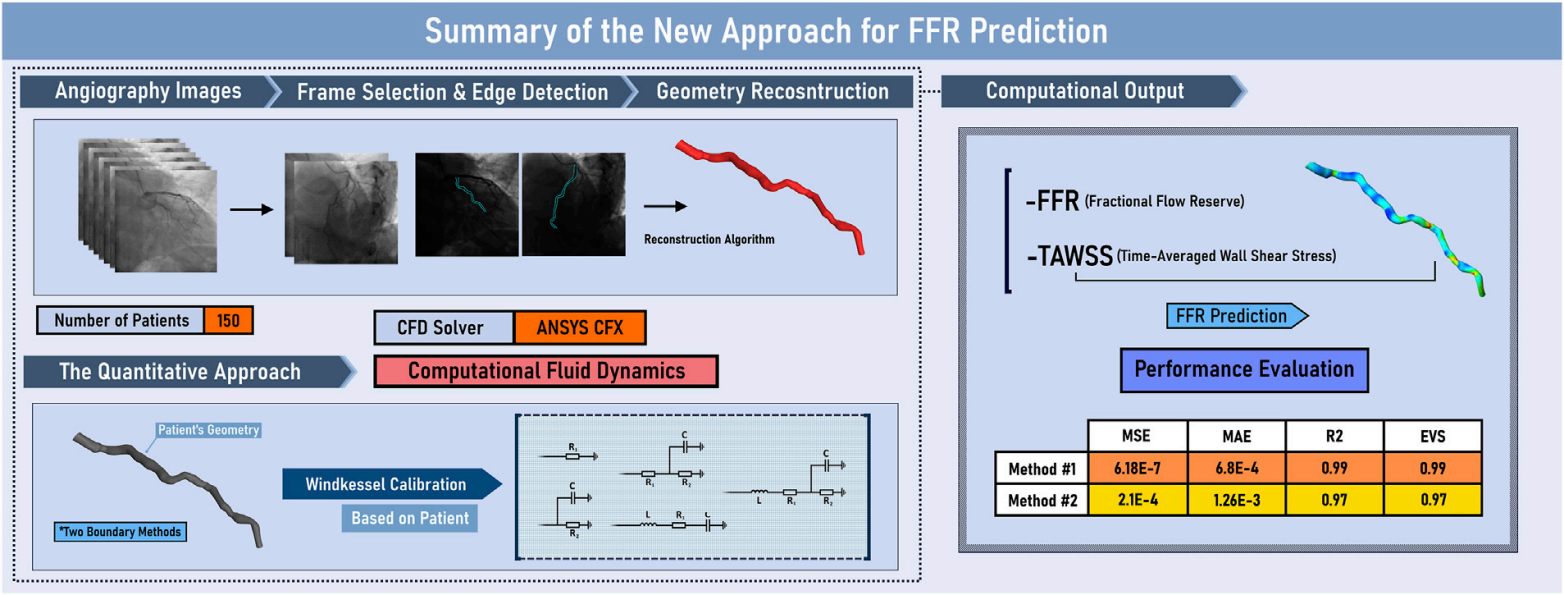
Graphical Abstract. EVS: explained variance score; FFR: Fractional Flow Reserve; MAE: mean absolute error; MSE: mean squared error; R2: R-squared; TAWSS: time-averaged wall shear stress.

Various methodologies have been examined for the optimization of FFR approximation, as shown in Figure 10. The most optimal technique has shown reliable results for all 150 patients with the MSE of 6.186e-7 and R2 of 0.99 (p = 0.00434) for the first approach and with the MSE of 2.188e-4 and R2 of 0.97 (p = 0.12715) for the second approach. This study is the first to investigate the applicability of QFR from a regression lens, which deviates from the conventional classification approach used in previous studies. This highlights the strength of the methodology, as it has shown superior performance compared to recent state-of-the-art approaches when translated into a classification approach. Furthermore, the current investigation offers important hemodynamic information for the prediction of future blockage or advance of the existing infarction, which is absent from other investigations. The results are both computationally and clinically user-friendly, with the accumulative pre and post-processing time of 15 min on a desktop computer (Intel i7 processor, 16 GB RAM). The proposed methodology has the potential to substitute wire-based FFR measurements, improving the evaluation of physiological coronary lesions in the catheterization laboratory and further assisting clinical diagnosis.

## Data Availability

The data analyzed in this study is subject to the following licenses/restrictions: The data are not publicly available due to privacy restrictions by the medical center. Requests to access these datasets should be directed to mona@alimohammadi.co.uk.
